# An Attention EfficientNet-Based Strategy for Bearing Fault Diagnosis under Strong Noise

**DOI:** 10.3390/s22176570

**Published:** 2022-08-31

**Authors:** Bingbing Hu, Jiahui Tang, Jimei Wu, Jiajuan Qing

**Affiliations:** 1 Faculty of Printing, Packaging Engineering and Digital Media Technology, Xi’an University of Technology, Xi’an 710048, China; 2 School of Mechanical and Precision Instrument Engineering, Xi’an University of Technology, Xi’an 710048, China

**Keywords:** attention mechanism, EfficientNet, fault diagnosis, rolling bearing

## Abstract

With the continuous development of artificial intelligence, data-driven fault diagnosis methods are gradually attracting widespread attention. However, in practical industrial applications, noise in the working environment is inevitable. This leads to the fact that the performance of traditional intelligent diagnosis methods is hardly sufficient to satisfy the requirements. In this paper, a developed intelligent diagnosis framework is proposed to overcome this deficiency. The main contributions of this paper are as follows: Firstly, a fault diagnosis model is established using EfficientNet, which achieves optimal diagnosis performance with limited computing resources. Secondly, an attention mechanism is introduced into the basic model for accurately establishing the relationship between fault features and fault modes, while improving the diagnosis accuracy in complex noise environments. Finally, to explain the proposed method, the weights and features of the model are visualized, and further attempts are made to analyze the reasons for the high performance of the model. The comprehensive experiment results reveal the superiority of the proposed method in terms of accuracy and stability in comparison with other benchmark diagnosis approaches. The diagnostic accuracy under actual working conditions is 86.24%.

## 1. Introduction

Prognostics and health management (PHM) is essential for modern industry [1,2]. However, the rotating machinery in the modern industry generally operates in a harsh working environment, and the mechanical transmission system would inevitably produce various types of failures, which may easily lead to accidents or economic losses [3,4,5]. Bearings are one of the important components in the transmission system, and their health has a direct impact on the performance and stability of mechanical equipment. Therefore, more accurate and smarter bearing health monitoring technology is extremely desirable for the stable working condition of rotating machinery [6,7,8].

As one of the advanced algorithms, deep learning has played an important role in computer vision, natural language processing, speech recognition, and other fields [9]. Recently, deep learning methods have also been widely used in the field of PHM [10]. Tang [11] discussed the existing fault diagnosis methods for rotating machinery. He also analyzed the future investigation direction of this field. Khemani [12] optimized the second-generation wavelet transform operators using a genetic algorithm, and established a fault diagnosis method for wavelet-scattering networks based on the optimization results. Wang [13] developed an approach based on the conditional variational auto-encoder generative adversarial network (CVAE-GAN) for the imbalanced data in the fault diagnosis of the planetary gearbox. Zhou [14] proposed a global optimization GAN, and the training process of this method is guided by fault feature and fault diagnosis error, so it has higher diagnostic accuracy than traditional generative models. Zhu [15] proposed an intelligent fault diagnosis method by combining principal component analysis (PCA) and deep belief network (DBN). The results indicate that this method can effectively achieve fault diagnosis of rolling bearing. He [16] developed a method for the weak fault diagnosis of bearing. This method uses fractional Fourier transform (FRFT) to transform the original signal into the fractional domain and performs filtering, and then uses deep belief networks (DBN) to adaptively extract the bearing fault features.

In addition to the above models, convolutional neural network (CNN) has been extensively studied for fault diagnosis. This method utilizes operations such as local receptive fields and weight sharing to extract fault features more quickly and accurately. With the continuous development of CNN, some excellent architectures have emerged, such as VGG, ResNet, Inception, etc. These algorithms have broad applications in fault diagnosis. Dibaj [17] proposed a fault diagnosis method that combines fine-tuned variational mode decomposition (VMD) and CNN to realize bearing compound fault diagnosis. A single fault sample was adopted to train the model during this process. Wen [18] put forward a fault diagnosis method for hierarchical convolutional neural network (HCNN), which trained two classifiers simultaneously for diagnosing fault patterns and fault severity. Huang [19] introduced a one-dimensional deep convolutional neural network (DCNN) in compound fault diagnosis. Zhang [20] provided a slope and threshold adaptive activation function with the tanh function, and ResNet was introduced to realize the fault diagnosis of rolling bearings. Zhang [21] used the channel attention mechanism to improve the feature extraction ability of the ResNet model. Chen [22] utilized the Inception v3 model to build a transfer learning model to realize the fault diagnosis of the wind turbine with imbalanced data. Wang [23] employed a multi-sensor model based on VGG and swarm decomposition for fault diagnosis, and the experimental results show that this method has good diagnostic accuracy and robustness.

However, it remains a most challenging issue to establish the excellent fault diagnosis model with limited circumstances [24]. The traditional approach is to continuously increase the depth or width of the model, which places high demands on the computer hardware and increases the difficulty of intelligent fault diagnosis [25,26]. Moreover, a great increase in the depth or width of the model limits the performance of the model, so a proper balance of width and depth remains a key issue to be addressed.

As an efficient classification algorithm, EfficientNet provides an amazing way to scale neural network models by enhancing depth, width, and resolution [27,28]. It is a CNN and scaling technique that applies compound coefficients to uniformly scale depth, width, and resolution dimensions. This process avoids the limitations of traditional convolutional neural networks on this problem.

For this purpose, a fault diagnosis model based on EfficientNet is proposed in this paper. Continuous wavelet transforms (CWT) [29] is employed to process bearing vibration signals to obtain time frequency representations (TFRs), and a new composite scaling method is utilized to balance the depth, width, and resolution of the model, which can improve the diagnostic accuracy in fault diagnosis and maximize resource utilization.

Meanwhile, it is worth noting that noise exists in the actual working environment of mechanical equipment. The collected signals during the acquisition process contain characteristic features of the fault, but this feature information might be submerged in much noise, leading the model to learn inaccurate features [30,31]. Hence, noise is a key factor affecting the accuracy of diagnostic models. Improving model efficiency under heavy background noise is critical for fault diagnosis. To this end, two attention mechanisms are introduced to capture the feature dependencies in the TFRs of faults. Specifically, the spatial attention mechanism and the channel attention mechanism are added to the model to emphasize the fault features in the TFR [32]. It can avoid the interference of noise areas. Bearing experiments are carried out to validate the proposed method, and the results indicate that this method outperforms other existing methods. A detailed analysis is also provided for the application of the proposed method in actual working conditions.

In this paper, a fault diagnosis method is proposed by combining time–frequency analysis and a new architecture, EfficientNet. The proposed method in this paper improves the efficiency of fault diagnosis with limited resources and also ensures diagnosis accuracy. In addition, the influence of background noise on the model accuracy is significantly reduced. The main contributions of this paper are summarized as follows.

(1) A fault diagnosis method for rolling bearings based on EfficientNet is proposed. Compared with other methods, this method can obtain the optimal diagnosis efficiency with limited resources.

(2) To reduce the influence of noise on diagnostic accuracy, a dual attention mechanism is introduced to refine the local features and effectively capture global features. The role of the attention mechanism is presented through the visualization of the results.

(3) The programmed algorithm is evaluated with noisy samples, and the diagnostic accuracy is 89.54%. In the application of actual working conditions, the model also is superior to other methods, and the diagnostic accuracy is 86.24%.

The rest of the paper is organized as follows: Section 2 presents the preliminaries of the proposed method for fault diagnosis in this paper. Section 3 explains the structure of the proposed fault diagnosis model. The experiments and results analysis are presented in Section 4. Finally, we draw a conclusion in Section 5.

## 2. Preliminaries

### 2.1. EfficientNet Architecture

To accurately extract representative fault features and build the optimal model with the limited computational resources, EfficientNet is selected in this paper as the baseline model to construct a rolling bearing fault diagnosis framework. The EfficientNet model is primarily constructed with MobileNet [33,34,35]. In contrast to traditional convolutional neural networks, the cores of MobileNet can be summarized in the following two parts.

#### 2.1.1. Depthwise Separable Convolution

Depthwise separable convolution (DSC) layers are used as the fundamental building blocks of MobileNet. Therefore, this structure is also known as the mobile convolutional (MB Conv) layer. This operation successfully reduces the computation latency and parameter size. The DSC is composed of two parts: depthwise convolution (DWC) and pointwise convolution (PWC).

The DWC is constructed with the same number of filters as the input channels, so it configures the corresponding filter for each channel. This ensures that the output image after passing through the layer still retains its depth. Specifically, DWC utilizes a single 3 × 3 convolution kernel to slide layer by layer over the input feature map, thereby generating an output channel after each slide.

The main function of PWC is to adjust the depth of the input feature map. The process is implemented by adjusting the thickness of the output with a 1 × 1 convolution kernel. The output with a single kernel is generated after the PWC operation.

**Figure 1:**
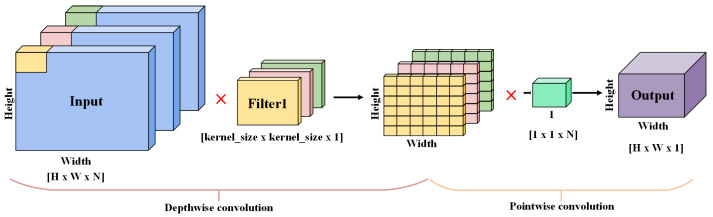
Depthwise separable convolution.

The convolution process with the combination of DWC and PWC is shown in Figure 1, which replaces the traditional convolution operation. The objective of lightweight model parameters while maintaining output quality is achieved.

#### 2.1.2. Inverted Residuals

It proves that residual blocks are helpful to build deeper networks with a strong performance in ResNet. For the same purpose, a similar block is introduced in MobileNet. Traditional residual blocks connect the convolutional layers using skip connection, where the start and end layers are wide and the middle layers are narrow. In other words, the features are first down-dimensioned, and then up-dimensioned after a convolution operation. However, inverted residuals are the opposite, where features are first up-dimensioned and then down-dimensioned after a convolution operation. This is also designed for DSC to proceed smoothly, ensuring that the feature extraction process can be carried out in a high-dimensional state.

#### 2.1.3. EfficientNet B2 Architecture

To capture rich features, there is a preference for increasing the width and depth of the model in traditional data-driven fault diagnosis investigations. Indeed, many theories and studies have demonstrated that larger models are easier to be trained and for capturing fine-grained features. However, as the network width continues to expand, its precision tends to saturate such that the model is no longer actively capturing advanced features. Similarly, increasing the depth of the model improves its ability to learn complex features, but it is prone to gradient disappearance and explosion. Furthermore, it is also difficult to balance the model accuracy and speed in terms of the selection of the model resolution. The EfficientNet uses MobileNet as a basic framework, which improves this problem. This approach searches for the optimal model with limited resources by scaling the depth, width, and resolution of the model. In contrast to the arbitrary design concepts of standard convolutional models, this method uses the composite coefficients ϕ to uniformly scale the width, depth, and resolution of the network. This developed compound scaling method can be briefly described in (1).

(1) $$\begin{array}{c} \begin{array}{cc} \; & Depth:d=\alpha^{\phi },\\ \; & Width:\omega =\beta^{\phi },\\ \; & Resoultion:r=\gamma^{\phi },\\ s.t.\;\; & \alpha \cdot \beta^{2}\cdot \gamma^{2}\approx 2,\\ \; & \alpha \geq 1,\beta \geq 1,\gamma \geq 1 \end{array} \end{array}$$

where ϕ is a coefficient used for controlling the scaling of the model, and the constants α,β,γ can be obtained by means of a small grid search. In a general convolution operation, the floating-point operations per second (FLOPS) are proportional to $d,\omega^{2},r^{2}$. FLOPS is raised to approximately ${\left(\alpha \cdot \beta^{2}\cdot \gamma^{2}\right)}^{\phi }$ by the scaling operation of Equation (1). To control the computational cost, the value of $\alpha \cdot \beta^{2}\cdot \gamma^{2}$ is set to 2, and the final FLOPS is $2^{\phi }$. Beginning with the baseline model EfficientNet-B0, the compound scaling method scales the model in two steps:

Step 1: Assuming that twice the available computational resources are currently available, the small grid search method is carried out under ϕ=1. The optimal parameters of EfficientNet B0 are finally optimized under the $\alpha \cdot \beta^{2}\cdot \gamma^{2}\approx 2$ condition, i.e., α=1.2, β=1.1, γ=1.15.

Step 2: According to Equation (1), EfficientNet B0 is scaled up to obtain versions B1 to B7 under different ϕ values.

Given the limited computing resources, EfficientNet-B2 pre-trained by ImageNet is adopted as the baseline structure of the model. More details are given in Section 2.3.

### 2.2. Attention Mechanisms

Since bearings operate under a variety of conditions, fault features are easily buried by complex background noise and interference. Background noise causes less variation between different levels of fault features of the same type. The traditional intelligent diagnosis methods are easily misled by noise when extracting fault features. Therefore, these factors seriously affect the diagnosis accuracy of intelligent fault models. It remains the key issues to enable the model to extract effective fault features without noise interference.

Currently, we have noted in existing research that the attention mechanism can increase the receptive field of the underlying features via the attention map. Hence, it can be implemented to emphasize representative features and suppress irrelevant information. This prompts the model to distinguish fault features from noise, thereby improving the diagnosis accuracy.

In this paper, an attention mechanism is introduced to the EfficientNet, which can not only increase the precision of fault feature extraction under complex background noise, but also adaptively integrate the dependencies between local and global features of fault samples.

The introduced location-wise soft attention includes channel and spatial attention mechanisms [32]. The channel attention mechanism mainly performs maximum pooling and mean pooling over the spatial extent of the feature graph, and then two different features are obtained to represent the information in the space. These features are then fed into a multi-layer perceptron to generate the corresponding channel attention maps. Finally, the resulting features are combined using multiplicative weighting, which can realize the sensitivity calibration of features in the channel dimension. The channel attention mechanism can be defined as follows:

(2) $$\begin{array}{c} \begin{array}{cc} M_{c}\left(F\right)= & \mathrm{Sigm}(MLP(\mathrm{Avgpool}(F))+(MLP(\mathrm{Maxpool}(F))) \end{array} \end{array}$$

where *F* denotes the input features, Sigm indicates the sigmoid function, and MLP represents the multi-layer perceptron.

The main role of spatial attention is complementary to channel attention. After feeding the feature map, the global features are initially extracted on the same channel of the feature map, which is achieved by performing maximum pooling and mean pooling operations, respectively. Afterward, these two features are concatenated, and a spatial feature map is generated using a convolutional layer. The above spatial attention operation implements the recalibration of features in spatial dimensions. It is calculated by

(3) $$M_{c}\left(F\right)=\mathrm{Sigm}\left(f_{7^{\times }7}\left(\left[\mathrm{Avgpool}\left(F\right);\mathrm{Maxpool}\left(F\right)\right]\right)\right)$$

where $f_{7\times 7}$ denotes a convolutional layer with a 7 × 7 kernel.

### 2.3. Attention EfficientNet

**Figure 2:**
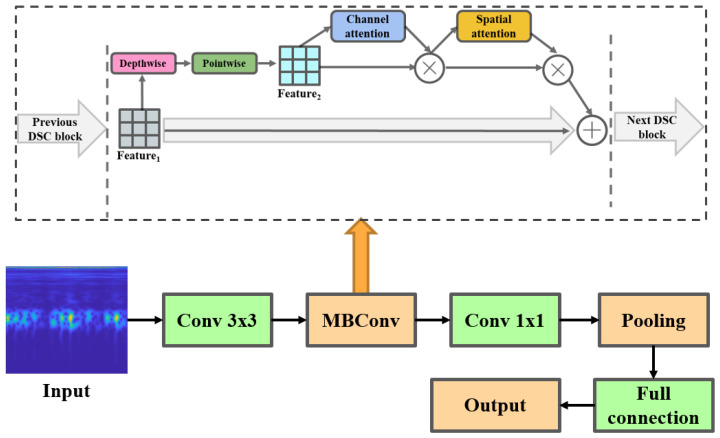
An overview of attention EfficientNet.

In this paper, the proposed attention EfficientNet is built using MobileNet as the basic module. The process decomposes the traditional convolution into two steps: depthwise convolution and pointwise convolution. This significantly reduces the number of weight parameters computed by the network and improves the computation speed. In addition, the conventional EfficientNet is modified to enhance the model ability to learn fault features. Location-wise soft attention is introduced to the standard model, which improves the sensitivity of the model to fault features under heavy background noise. A *softmax* associated with the actual fault classes is added to the head of the subsequent network for classification. Figure 2 illustrates the proposed model. Table 1 displays the network structure of attention EfficientNet.

## 3. Attention EfficientNet-Based Fault Diagnosis Framework

**Figure 3:**
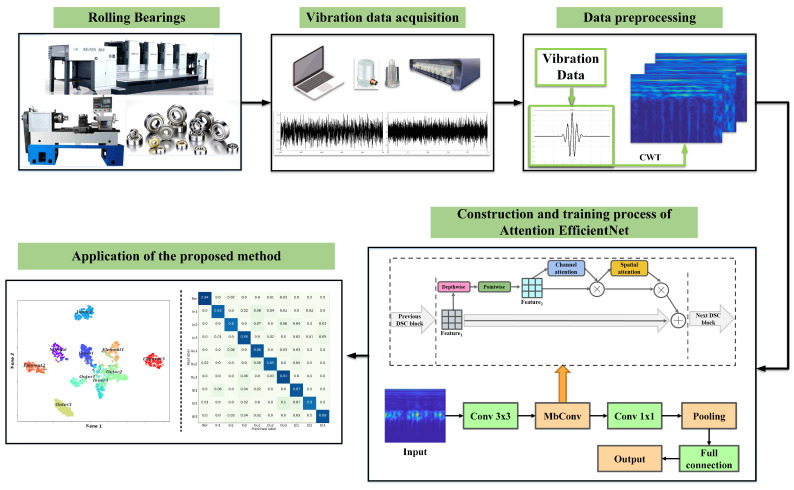
General diagnosis procedure of attention EfficientNet.

To improve the efficiency of the intelligent fault diagnosis model, as well as improving the diagnosis accuracy of the model when the samples have complex noise, an intelligent fault diagnosis method based on EfficientNet and the attention mechanism is proposed in this paper. Specifically, Figure 3 illustrates the general diagnosis procedure of the present method, and the detailed steps are described as follows.

(1) The vibration signals of the rolling bearing are collected by the acquisition equipment.

(2) The TFRs are obtained by transforming the original vibration signals via CWT, and then they are labeled as training samples.

(3) The hyperparameters and structural parameters of the attention EfficientNet model are initialized.

(4) The fully trained model is applied to identify the test samples.

(5) The performance of the proposed model is finally evaluated by the diagnosis results.

## 4. Experiment Analysis

In practical engineering applications, the operating environment of the mechanical system is complicated, and noise interference is a primary driver to contaminate the vibration signals of rolling bearings. It is a challenging task to improve the model performance for fault diagnosis under strong noise. The proposed method in this paper is developed to address this situation.

Therefore, the effectiveness of the proposed method is evaluated by setting up experiments containing different levels of background noise in this section. In addition, the effectiveness of the proposed method is verified using an operational printing equipment test bench, which is designed to evaluate the model efficiency under actual working conditions.

### 4.1. Case 1: Performance under Simulation Noise Environment

#### 4.1.1. Data Description

**Figure 4:**
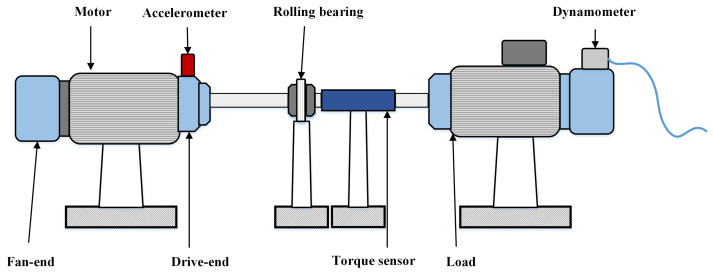
The bearing experimental setup of CWRU.

The effectiveness of the proposed method under simulation noise is investigated in this section. This experiment uses data provided by Case Western Reserve University Lab (CWRU) [36], which are obtained from measurements of the motor-driven mechanical system. The basic structure of the bearing experimental setup is illustrated in Figure 4. The experimental bearing contains four categories of fault—inner ring fault, outer ring fault, rolling element fault, and normal condition—and each condition contains three fault diameters. The sampling frequency is 12 kHz. Each sample contains 1000 sample points, which are later processed into a TFR using CWT and used as input to the model. More details on the dataset are outlined in Table 2.

Considering the highly variable noise, we fail to obtain enough labeled samples under different noise conditions. To simulate complex noise scenarios in real industrial production, a composite signal is formed by combining different signal-to-noise ratios (SNRs) with the original signal with additive white Gaussian noise. These composite signals are adopted to validate the diagnosis performance of the attention EfficientNet under different strong noise. The SNR is defined as below.

(4) $$SNR_{\mathrm{dB}}=10\log_{10}\left(\frac{P_{\mathrm{signal}\;}}{P_{\mathrm{noise}\;}}\right)$$

where $P_{\mathrm{signal}\;}$ and $P_{\mathrm{noise}\;}$ the power of the noise and signal respectively.

Although the raw vibration data contain sufficient information about the bearing health condition, it is not clear the interpretability of directly applying this information to fault diagnosis. Considering the non-stationarity of the vibration signal under actual working conditions, the continuous wavelet transform is utilized to process the raw data in this paper.

According to existing investigations, it can be found that Morlet wavelets have a low error rate for extracting periodic shock signals from bearing vibration signals. In addition, the time–domain waveform of the Morlet wavelet is similar to the impact features of the bearing fault signal, which allows a well-matched relationship between it and the fault signal. However, the resolution of TFR directly processed via CWT reaches 1167 × 875; this undoubtedly increases a huge amount of model structure parameters and computational consumption. For this purpose, a bicubic interpolation algorithm is adopted to resize the original image to 260 × 260 (common size). These TFRs can be used as input to the attention EfficientNet.

**Figure 5:**
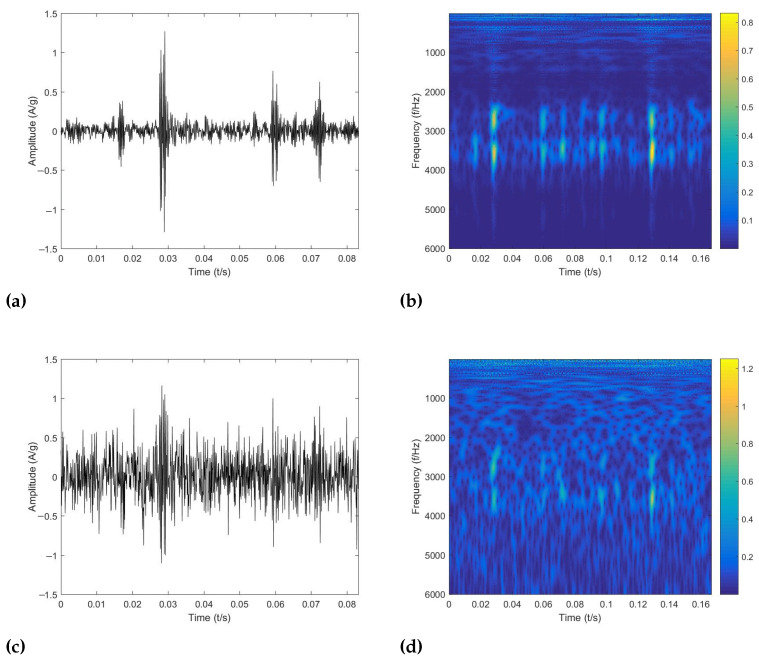
The analyzed results with CWT. (**a**) Vibration signal of inner race fault. (**b**) TFR of inner race fault. (**c**) Vibration signal of inner race fault with additive noise. (**d**) TFR of inner race fault with additive noise.

Figure 5 shows the original signal and the composite signal with the addition of Gaussian white noise of the inner ring fault, and it also illustrates the corresponding TFR. Figure 5 shows the signal distribution at an SNR of −4 dB, i.e., the ratio of the power of the pure signal to the power of noise is −4. As can be seen from the figure, the added noise makes the fault features in the TFR unclear, which may have a significant impact on the performance of conventional intelligent diagnosis methods.

#### 4.1.2. Baseline Approaches

To verify the validity of the proposed method, we use the standard EfficientNet as a comparison method. In addition, we compare the proposed method with ResNet [37]. ResNet uses residual blocks to achieve cross-layer connections between network layers, increasing the depth of the model while avoiding performance degradation. Similarly, the visual geometry group (VGG) [38] model and the Inception-v3 [39] model have superior performance in fault diagnosis, which are also chosen as comparison methods.

The above comparison methods are designed with the same input dimensions as the present method, and *softmax* is chosen as the classifier. Meanwhile, corresponding ablation experiments are added to each method for investigating the role of attentional mechanisms.

#### 4.1.3. Model Selection

The proposed method is written based on the deep learning framework Pytorch, and the experiments were carried out on a Core i7-9700, NVIDIA RTX 2070. The model hyperparameters in this study are all obtained from cross-validation experiments, with an initial learning rate of 0.01, a batch size of 4, and the number of iterations set to 30. To minimize cross-entropy losses, a stochastic gradient descent (SGD) optimizer with 0.9 parameter momentum is introduced. In addition, a dropout with a scale of 0.3 is introduced to moderate the overfitting phenomenon.

The validity of the model is tested with composite signals SNR, ranging from −6 dB to 6 dB. In the process, we simultaneously demonstrate the necessity of the small-batch training sample option. The batch sizes are established to the integer power of 2, and this is to conform to the coding patterns of the computer and enhance operational efficiency. The parameters remain the same for each experiment, except for the SNR and batch size. The results are displayed in the Table 3.

It is evident from the table that the diagnosis accuracy declines as the SNR decreases regardless of batch size. When the SNR is −6 dB, the diagnosis accuracy of the model is lower than in the other cases. However, as the SNRs increase, diagnosis accuracies of the models also increase. For instance, when the SNR is larger than 4 dB, the diagnosis accuracy generally exceeds 95%. These situations are attributed to the fact that noise enhances the difficulty of the model in feature learning, thereby reducing the diagnosis accuracy. In contrast, small batch sizes of learning patterns outperform large batch sizes for the same SNRs. When the SNR is −4 dB, the diagnosis accuracy of a batch size of 4 is approximately 4.5% higher than the batch size of 128. It can be concluded that a small batch size learning pattern is helpful for the model to extract more accurate fault features.

#### 4.1.4. Results and Analysis

**Figure 6:**
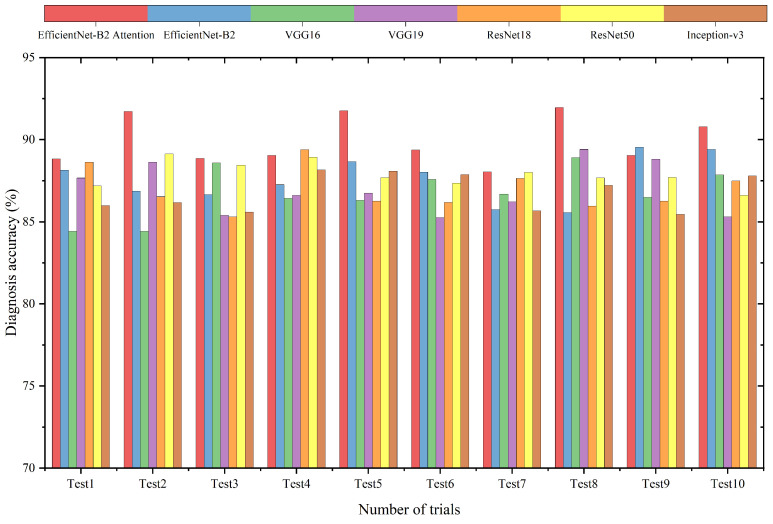
Detailed experimental results of different methods.

Based on the results in the previous section, the optimal hyperparameters are determined for the attention EfficientNet. In this section, the comparison experiments are performed on the composite signal dataset. It is worth mentioning that the SNR is −4 dB in this case. Figure 6 and Table 4 show the detailed and average results of the 10 trials of the proposed method, respectively. From the figure and the table, it is observed that the accuracy of attention EfficientNet (89.54%) is higher than other methods. The accuracy variance is also lower than other methods, which indicates that the results of the proposed method are more stable. It also can be noted that the attention mechanism provides the model with an accuracy improvement of approximately 2% (standard EfficientNet: 87.58%).

In addition, we found that the deep model achieved better results than the shallow model. For example, the average accuracy of ResNet50 is 87.87%, while the accuracy of ResNet18 is 87.06%. Additionally, the accuracy variance indicates that ResNet50 is more stable. Compared to other methods, the Inception architecture reduces the number of model parameters and improves the performance degradation problems caused by overfitting. Although the improvement in accuracy is limited, results reveal that this approach enhances the stability of the model.

**Figure 7:**
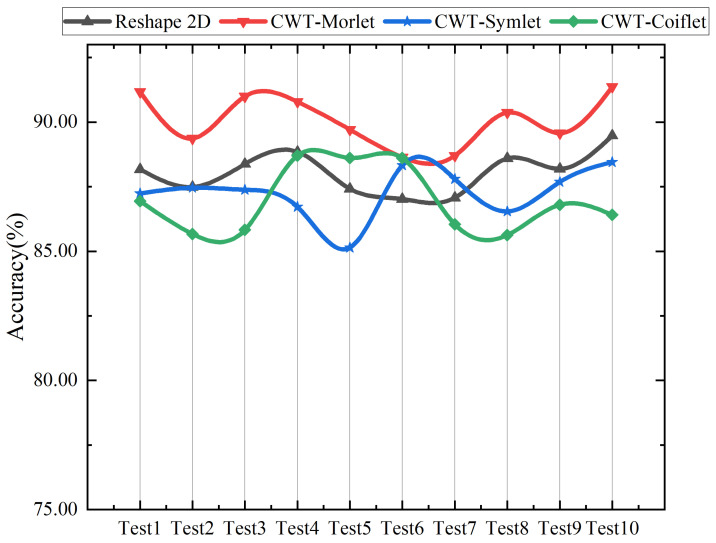
Detailed experimental results under different pre-processing methods.

The diagnosis results of the attention EfficientNet with different pre-processing methods are shown in Table 5 and Figure 7. Three commonly used wavelets are employed to generate TFRs, and the original signal is directly truncated and converted to a two-dimensional signal as a control experiment. From the results, the model processed by the Morlet wavelet has the superior diagnostic performance.

#### 4.1.5. The Necessity of Attentional Mechanisms

The EfficientNet fault diagnosis method proposed in this paper is based on the attention mechanism to extract more representative features from TFR. Hence, it is necessary to employ attention visualization to explore the role of this mechanism. The main purpose of this mechanism employed in the proposed method is to enable the model to increase the emphasis on representative features in the TFR. Specifically, the attention mechanism is adapted to adjust the distribution of features during feature extraction, which enables the model to focus on local features and reduces the effect of noise.

**Figure 8:**
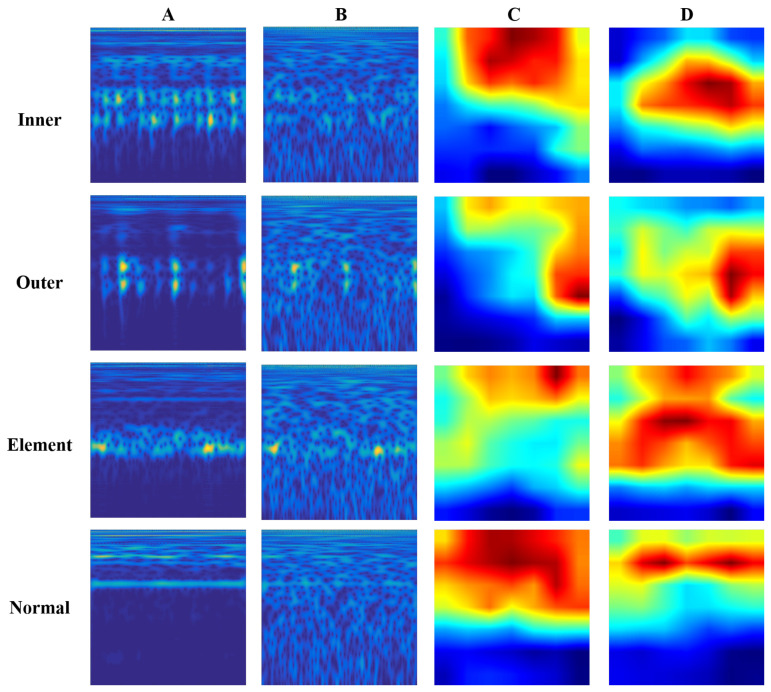
The attention weight visualization of proposed method. (**A**) Original TFRs; (**B**) TFRs with additional noise; (**C**) weight distribution of EfficientNet; (**D**) weight distribution of attention EfficientNet.

In this context, we attempt to show the degree of attention for attention EfficientNet using the attention weights. As shown in Figure 8, this section displays the TFR of the original signal and the composite signal (input samples). Moreover, the attention weight distribution in the last convolutional layer of the standard EfficientNet and the proposed method are also shown.

It is clear from the Figure 8 that the standard EfficientNet has endowed the model with a better learning strategy, as the weights show a tendency to focus on the fault features. It is even more evident with the attention mechanism that the network assigns different weights to different fault types. To sum up, the EfficientNet has more concentrated weights on the convolutional layer with this attention mechanism, and the fault feature components are comprehensively covered. The ability to focus on the fault feature components helps the model extract the representative features of the different fault types and avoids the influence of irrelevant factors, such as noise. Fault feature components are more comprehensive and can focus on representative features. Thus, attention EfficientNet enables the model to extract representative features of different fault types without the interference of noise.

### 4.2. Case 2: Performance under a Realistic Noise Environment

#### 4.2.1. Data Description

**Figure 9:**
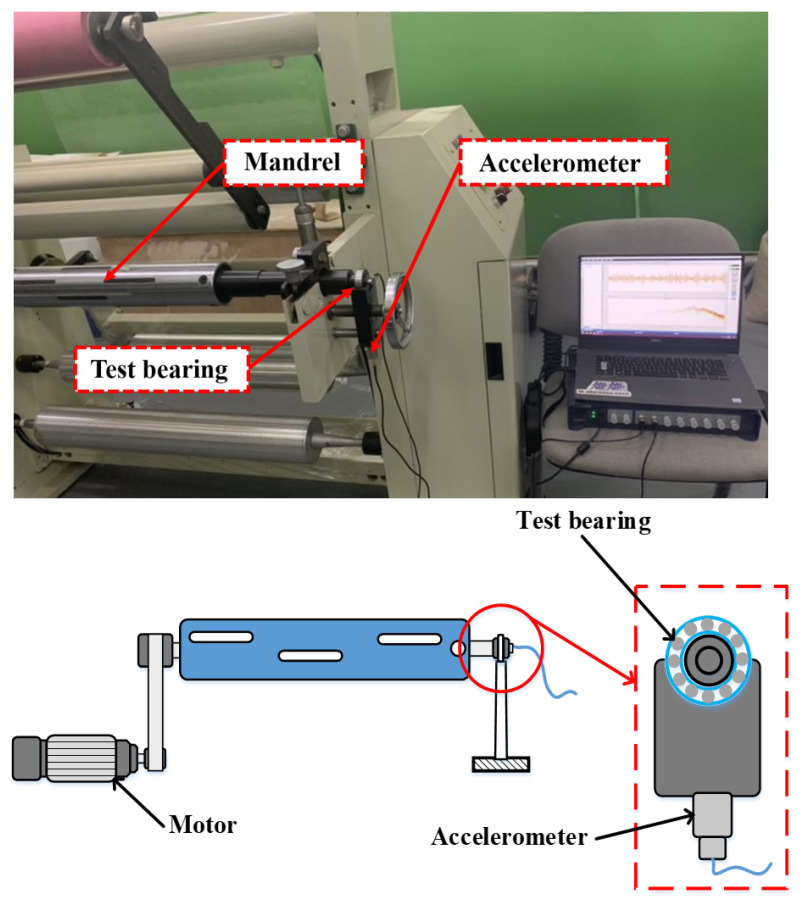
The bearing test rig of press mandrel.

**Figure 10:**
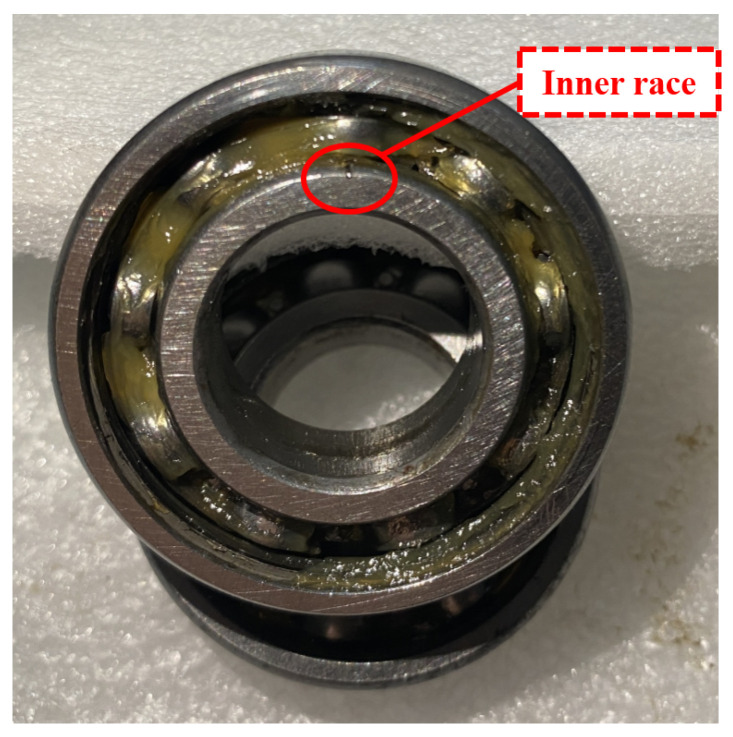
The test bearing with inner race fault.

**Figure 11:**
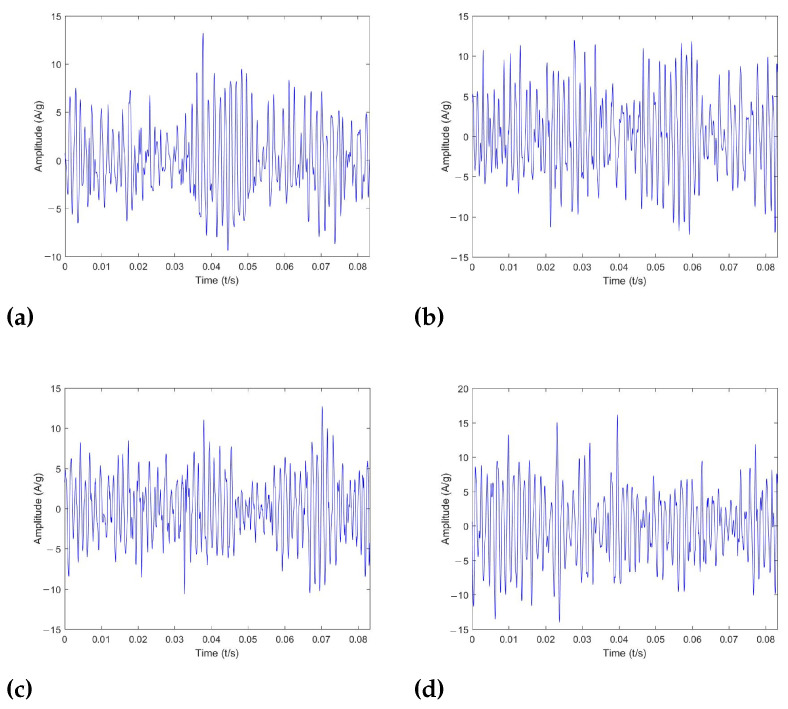
Examples of experimental signals. (**a**) Normal; (**b**) inner race fault; (**c**) outer race fault; (**d**) cage fault.

To verify the effectiveness of the proposed method in real-world noise, this method is examined on typical rotating equipment (printing press) with actual conditions. Figure 9 shows the experimental platform. The bearings under test are mounted at both ends of the mandrel, and the device is driven by a separate servo motor with the speed controlled via a frequency converter. The experimental bearing type is the deep groove ball bearing and its parameters are shown in Table 6. Figure 10 shows an inner ring bearing fault (diameter 0.4 mm) introduced by electrical discharge machining (EDM); the other two forms of failure are outer ring failure and cage failure. Each fault type contains three diameters of 0.2 mm, 0.4 mm, and 0.6 mm. Accelerometers with a sensitivity of 100 mv/g are mounted on the bottom and side of the bearing housing to measure the vibration signals, and the sampling frequency is 12 kHz. A selection of samples from this dataset is illustrated in Figure 11, from which it can be seen that the periodic impact features of rolling bearing failures are contaminated by interference of noise.

The detailed information of datasets is shown in the Table 7. The procedure of data pre-processing used in this section is the same as in Section 4.1. To ensure the comprehensive evaluation of model performance, the dataset is randomly divided in each training and testing session.

#### 4.2.2. Diagnosis Results

In this section, the same comparison methods are selected as in Section 4.1. These methods also use crossover experiments to determine the hyperparameters. Based on the optimal model structure and hyperparameters, attention EfficientNet is trained on a printing press bearing dataset. The trained model is used to diagnose testing data to evaluate the diagnosis performance. Training and test are repeated over 10 trials on attention EfficientNet and six benchmark approaches. Table 8 illustrates the average accuracy and accuracy variance for this experiment. Compared to the results in Section 4.1, the diagnosis accuracy of all methods drops significantly. The reason is that the noise in real-world conditions is more complex, which makes diagnosis models more complicated to establish a relationship between fault features and fault categories.

However, the proposed method has a relatively satisfactory performance with an average diagnosis accuracy of 86.24%. Additionally, the accuracy variances reveal that the proposed method has the best stability in real-world noise environments compared to other methods.

**Figure 12:**
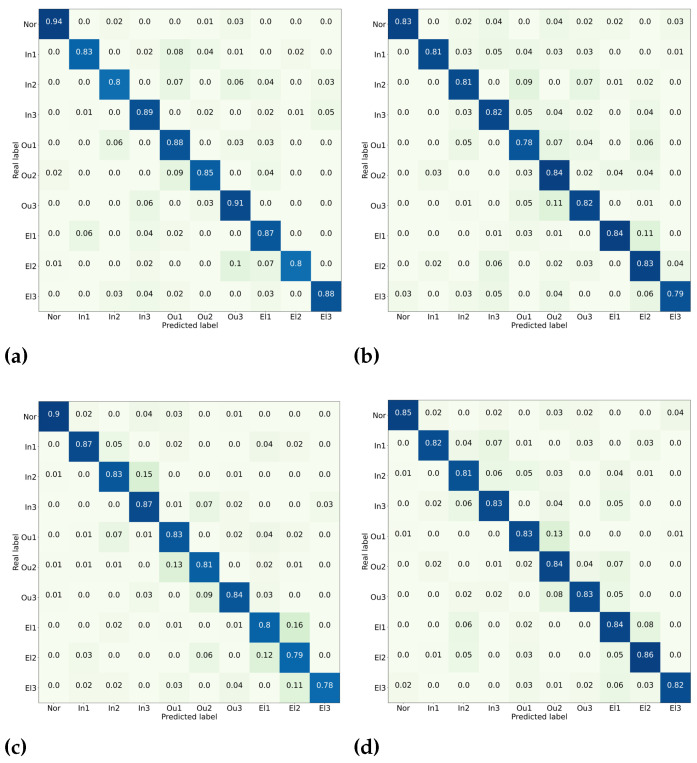
Confusion matrices for different methods. (**a**) Proposed method; (**b**) VGG19; (**c**) ResNet50; (**d**) Inception-v3.

The confusion matrices for four methods in the third experiment are given in Figure 12. In the confusion matrix, the rows represent the actual labels for the corresponding fault categories, and the columns represent the predicted labels. From the figure, it is obvious that the diagnostic accuracy of the proposed method for each fault type is generally higher than the corresponding types of other methods. Moreover, the accuracy rate exceeds 80% for each fault type. This result demonstrates the superior performance of the attention EfficientNet for multi-category fault identification under actual working conditions. It is apparent from Figure 12a that the proposed method is not optimal for diagnosing inner ring faults and cage faults, and the accuracy is approximately 80%. This result is inferior to the average diagnosis accuracy of the method, which indicates that both types are still more challenging to diagnose.

#### 4.2.3. Visualization of Feature Learning

**Figure 13:**
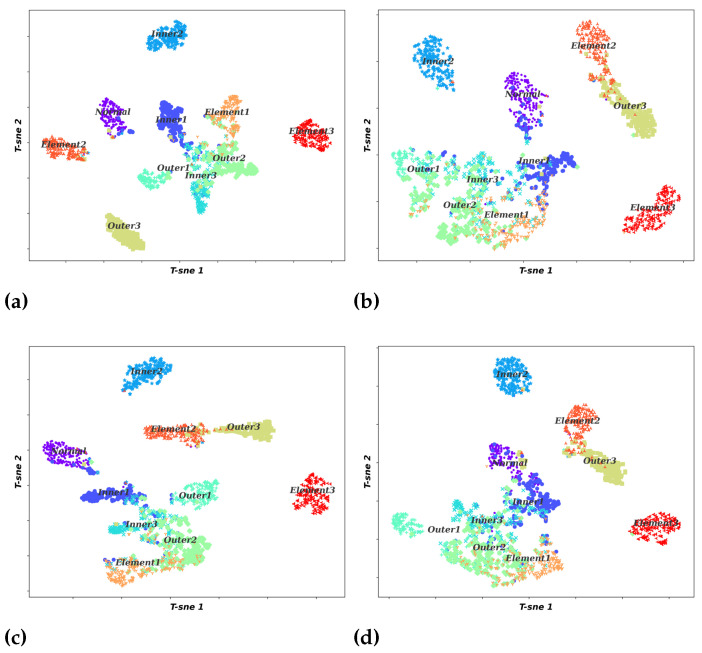
Two-dimensional visualization of the learned features by t-SNE. (**a**) Proposed method; (**b**) VGG19; (**c**) ResNet50; (**d**) Inception-v3.

To visualize the effect of the feature extraction process for different models, the high-dimensional features are displayed by the t-distributed stochastic neighbor embedding (t-SNE). In detail, this algorithm converts the high-dimensional features of the last fully connected layer of the four comparison methods into a two-dimensional distribution for visualization. The visualization results are shown in Figure 13. The figure reveals that most of the feature points in the four methods show a trend toward clustering. However, in the other three comparison methods, there is a more serious overlap of feature points, which is because the model is not yet accurate enough for representative feature learning. On the contrary, the proposed method has a more obvious clustering trend of feature points and less overlap. The results verify the validity of the proposed method for representative feature extraction.

## 5. Concluding Remarks

In this paper, a method based on attention EfficientNet is proposed to achieve the fault diagnosis of rolling bearing. The bearing vibration signal is first processed by CWT, and the multi-channel TFR is obtained. Next, these TFRs are then input into the MobileNet-based EfficientNet to extract hidden features for fault identification. In this process, the attention mechanism is introduced to guide the feature extraction process of the model, which enables the model to focus on the representative fault features and improves the diagnostic accuracy under complicated noise.

The effectiveness of the proposed method is verified by two experimental cases. Compared with other advanced methods, this diagnostic method has the following advantages: (1) This method can build an optimal model with limited resources, and the introduced attention mechanism improves the ability of the model to focus on fault features. (2) Compared with other methods, the proposed method has better diagnostic accuracy and stability. (3) This method has excellent performance under strong noise, and the average accuracy is 89.54% in the dataset containing simulated noise and 86.24% in the dataset containing real noise.

There is still much room for improvement, and our future work will focus on the following aspects. (1) More efficient signal preprocessing techniques will be explored to further improve the diagnostic accuracy of the model. (2) Different attention mechanisms are introduced to improve the feature extraction capabilities of the model.

## Figures and Tables

**Table 1:** Overall structure of attention EfficientNet.

Stage	Operator	Resolution	Channels	Layer
1	Input	260 × 260	3	1
2	Conv 3×3	130 × 130	35	1
3	Attention MBConv1 k3×3	130 × 130	18	1
4	Attention MBConv6 k3×3	65 × 65	26	2
5	Attention MBConv6 k3×3	32 × 32	44	2
6	Attention MBConv6 k3×3	16 × 16	88	4
7	Attention MBConv6 k3×3	16 × 16	123	4
8	Attention MBConv6 k3×3	8 × 8	211	5
9	Attention MBConv1 k3×3	8 × 8	352	1
10	Conv 1×1	8 × 8	1408	1
11	Pooling &FC	8 × 8	1408	1
12	Output	10 × 1	1	1

**Table 2:** Distribution of rolling bearing dataset.

Bearing Working Condition	Fault Diameter	The Number of Samples
Normal	—	200
Inner race fault	0.1778	200
	0.3556	200
	0.5334	200
Outer race fault	0.1778	200
	0.3556	200
	0.5334	200
Element fault	0.1778	200
	0.3556	200
	0.5334	200

**Table 3:** The accuracy of attention EfficientNet under different SNR and batch size.

Batch Size	SNR (dB)						
	−6	−4	−2	0	2	4	6
4	87.87%	89.86%	93.77%	95.91%	97.93%	97.95%	99.48%
8	87.78%	89.76%	93.26%	95%	95.21%	97.75%	99.31%
16	86.63%	88.52%	92.05%	94.08%	95.06%	97.62%	98.13%
32	86.3%	88.3%	91.46%	93.88%	94.8%	96.26%	98.19%
64	84.89%	88.07%	90.67%	93.56%	94.45%	95.49%	96.8%
128	83.39%	85.27%	90.41%	93.11%	94.34%	95.09%	96.68%

**Table 4:** Performance of different comparison methods.

Approach	Input Type	Diagnosis Accuracy	Accuracy Variance
Attention EfficientNet	CWT	89.54%	1.03
EfficientNet		87.58%	1.33
VGG16		86.76%	1.45
VGG19		87.00%	1.47
ResNet18		87.06%	1.39
ResNet50		87.87%	1.34
Inception-v3		86.80%	1.01

**Table 5:** The diagnosis results under different pre-processing methods.

Approach	Input Type	Diagnosis Accuracy	Accuracy Variance
Attention EfficientNet	Reshape 2D	88.07%	0.77
	CWT-Morlet	89.54%	1.18
	CWT-Symlet	87.27%	0.92
	CWT-Coiflet	86.93%	1.20

**Table 6:** Bearing parameters.

Parameter Description	Value
Bearing specs	6004
Bearing type	Deep groove ball bearing
Inner race diameter	20 mm
Outer race diameter	42 mm
Bearing thickness	12 mm
Roller number	9
Contact angle	0°
Bearing weight	0.069 kg

**Table 7:** Details of rolling bearing operation conditions.

Bearing Working Condition	Fault Diameter	The Number of Samples
Normal	—	300
Inner race fault	0.2	300
	0.4	300
	0.6	300
Outer race fault	0.2	300
	0.4	300
	0.6	300
Cage fault	0.2	300
	0.4	300
	0.6	300

**Table 8:** Performance of different comparison methods.

Approach	Input Type	Diagnosis Accuracy	Accuracy Variance
Attention EfficientNet	CWT	86.24%	0.59
EfficientNet		83.17%	0.77
VGG16		81.99%	0.93
VGG19		82.09%	0.61
ResNet18		81.87%	0.61
ResNet50		82.18%	1.07
Inception-v3		82.24%	0.76

## Data Availability

Not applicable.
